# ^68^Ga-FAPI-04 PET for Detecting Occult Peritoneal Metastasis in Locally Advanced Gastric Cancer: Diagnostic Performance and Cost Analyses in a Single-Center, Prospective Cohort Study

**DOI:** 10.2967/jnumed.125.270633

**Published:** 2026-01

**Authors:** Qiancheng Hu, Shunyu Zhang, Kun Yang, Yuan Yin, Xiaolong Chen, Mojin Wang, Bo Zhang, Wen Zhuang, Ming Liu, Chaoyong Shen, Pengfei Zhang, Hongyuan Dai, Junjun Cheng, Shuming Ji, Minggang Su, Hongfeng Gou, Jiankun Hu

**Affiliations:** 1 Department of Medical Oncology, Cancer Center, West China Hospital, Sichuan University, Chengdu, China;; 2 Gastric Cancer Center, West China Hospital, Sichuan University, Chengdu, China;; 3 Department of General Surgery, West China Hospital, Sichuan University, Chengdu, China;; 4 Department of Nuclear Medicine, West China Hospital, Sichuan University, Chengdu, China; and; 5 Department of Clinical Research Management, West China Hospital, Sichuan University, Chengdu, China

**Keywords:** ^68^Ga-FAPI-04 PET/CT, gastric cancer, occult peritoneal metastasis, laparoscopic staging

## Abstract

Occult peritoneal metastasis (OPM) is common in locally advanced gastric cancer, and accurate detection is critical. This prospective cohort study evaluated the diagnostic accuracy and cost of ^68^Ga-FAPI-04 PET/CT for detecting OPM. **Methods**: This single-center, prospective cohort study included patients with locally advanced gastric adenocarcinoma. All patients underwent ^68^Ga-FAPI-04 PET/CT before laparoscopic staging, and the diagnosis of OPM was established using laparoscopic staging combined with peritoneal washing cytology as the gold standard. The primary endpoint was the proportion of patients whose treatment intent changed based on ^68^Ga-FAPI-04 PET/CT results. Secondary endpoints included diagnostic accuracy and cost analysis of ^68^Ga-FAPI-04 PET/CT in detecting OPM. **Results**: In total, 109 patients were recruited between November 2022 and August 2024. ^68^Ga-FAPI-04 PET/CT identified OPM in 17 patients (15.6%), resulting in upstaging to stage IV, with sensitivity, specificity, and diagnostic accuracy of 75.0%, 94.6%, and 91.7%, respectively (area under the curve, 0.83; 95% CI, 0.72–0.94). Economic analysis demonstrated a net cost savings of $979.30 per patient when compared with laparoscopic staging. The combination of ^68^Ga-FAPI-04 PET/CT and laparoscopic staging reduced the need for laparoscopic procedures by 84% and prevented 11% of futile gastrectomies, yielding a minimal cost savings of $232.30 per patient. **Conclusion**: ^68^Ga-FAPI-04 PET/CT demonstrates high diagnostic accuracy, low cost, and the potential to reduce invasive procedures, making it a promising alternative to laparoscopic staging in patients with locally advanced gastric cancer.

## Visual Abstract

**Figure:**
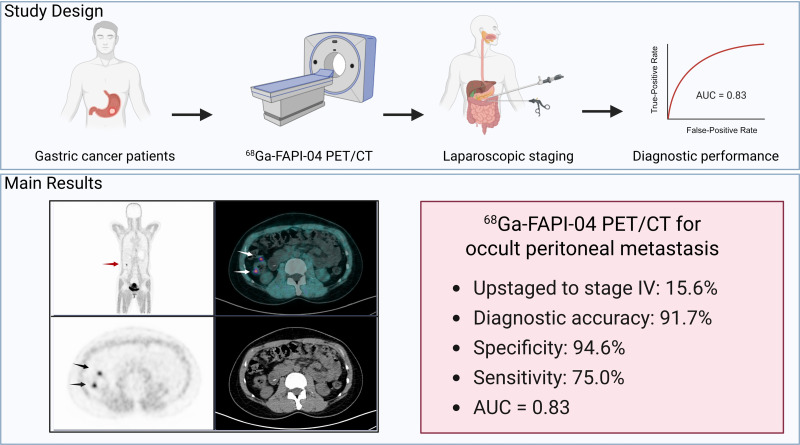

Gastric cancer represents a major global public health concern (1), particularly in East Asia, where it accounts for more than 40% of the worldwide occurrences (2). In China, most patients with gastric cancer are diagnosed at an advanced stage, leading to a 5-y survival rate lower than 30% (3). Peritoneal metastasis is the most frequent metastasis pattern in gastric cancer. Unfortunately, the therapeutic efficacy remains limited and the prognosis is poor (4). Accurate preoperative diagnosis of peritoneal metastasis is essential for guiding treatment decisions and preventing unnecessary gastrectomies.

Enhanced high-resolution CT scan can detect peritoneal metastasis in patients with characteristic imaging findings, such as omental caking, significant peritoneal thickening, large peritoneal nodules, or massive ascites. However, the diagnostic sensitivity of CT for peritoneal nodules with a small volume and low volume density is only about 25% (5). Peritoneal metastases not initially detected on CT scans but identified during laparoscopic exploration or open surgery are classified as clinically occult peritoneal metastasis (OPM) (6). In fact, OPM is a relatively common in patients with locally advanced gastric cancer, with a reported incidence of approximately 20% (5,7,8).

Laparoscopic staging, considered as the gold standard, is recommended for detecting OPM in patients with locally advanced gastric cancer. However, several disadvantages of laparoscopic staging, including 2-dimensional evaluation, high cost, limited ability to detect hepatic metastases and perigastric lymph nodes, and potential complications (e.g., trocar injury, port-site metastasis, immunologic compromise) (8), have hindered its widespread adoption in daily clinical practice.

Fibroblast activation protein is an endopeptidase primarily overexpressed by cancer-associated fibroblasts in the tumor stroma of gastric cancer (9,10). Gastric cancer demonstrates abundant fibroblast activation protein and intermediate ^68^Ga-FAPI-04 uptake (11). It addresses the limitations of PET/CT with ^18^F-FDG, which shows low avidity in diffuse and mucinous histologic cancer types but high physiologic uptake in the gastrointestinal tract (12,13). Growing evidence highlights the value of ^68^Ga-FAPI-04 PET/CT in detecting primary lesions, peritoneal metastasis, lymph node metastasis, and local recurrence in gastric cancer (13–15). However, the evidence supporting ^68^Ga-FAPI-04 PET/CT for diagnosing peritoneal metastasis of gastric cancer remains limited, as most studies rely on subgroup analysis or retrospective analyses that lack laparoscopic staging combined with pathologic biopsy as the gold standard for OPM (16,17). In addition, cost analyses of ^68^Ga-FAPI-04 PET/CT for detecting OPM in locally advanced gastric cancer are lacking. This study aimed to evaluate the diagnostic accuracy and cost of ^68^Ga-FAPI-04 PET/CT for detecting OPM in locally advanced gastric cancer.

## MATERIALS AND METHODS

### Study Design

This prospective, observational, single-center cohort study was conducted at West China Hospital in accordance with the ethical principles of the Helsinki II Declaration. The study was approved by the Ethics Committee of West China Hospital, and written informed consent was provided by all patients before ^68^Ga-FAPI-04 PET/CT. This study was registered in the Chinese Clinical Trial Registry (ChiCTR2300067591), and the study protocol was published elsewhere (18). Additional study methods are described in the supplemental materials, available at http://jnm.snmjournals.org) (19–22).

### Participants

We enrolled patients age 18 y or older with an Eastern Cooperative Oncology Group performance status of 0 to 1. Eligible patients had histologically confirmed gastric or gastroesophageal junction (Siewert type III) adenocarcinoma and were diagnosed with advanced gastric cancer (cT4a-b, N0–3, M0) using contrast-enhanced CT, according to the 8th edition of the American Joint Committee on Cancer staging system. All patients were scheduled to undergo curative-intent surgery in accordance with national guidelines. Exclusion criteria included suspected distant metastatic gastric cancer on CT staging, allergic constitution, and a history of abdominal inflammatory diseases (e.g., peritonitis, pancreatitis, cholecystitis, inflammatory bowel disease). Patients with contraindications to laparoscopic staging were also excluded.

### Procedures

All potentially eligible patients were discussed during the gastric cancer multidisciplinary team meeting. Consenting patients underwent ^68^Ga-FAPI-04 PET/CT within 14 d before laparoscopic staging. ^68^Ga-FAPI-04 PET/CT images were reviewed before laparoscopic staging by 2 board-certified nuclear medicine physicians, and any dissenting opinions were resolved through discussion. Surgeons were masked to the preoperative ^68^Ga-FAPI-04 PET/CT imaging results. Laparoscopic staging was performed using the “Huaxi 4-step” procedure, which has been used at our center for many years (23). During surgery, approximately 500 mL of peritoneal lavage fluid was routinely collected after laparoscopic exploration and immediately submitted for cytologic examination. Laparoscopic staging combined with peritoneal washing cytology served as the reference standard for the final diagnosis, and histopathologic examination of peritoneal implants was performed when indicated.

Data were collected using study-specific data collection forms, starting at recruitment. All data were stored electronically in a secure database, and strict confidentiality was maintained.

### Outcomes

The primary outcome was the proportion of eligible patients in whom ^68^Ga-FAPI-04 PET/CT altered the treatment strategy from curative to palliative intent treatment. The secondary outcomes included the following: evaluation of the diagnostic accuracy of ^68^Ga-FAPI-04 PET/CT for OPM, such as sensitivity, specificity, accuracy, positive predictive value, negative predictive value, positive likelihood ratio, and negative likelihood ratio in patients with locally advanced gastric cancers; assessment of peritoneal carcinomatosis lesion detectability using SUV_max_ and target-to-background ratio (TBR), where TBR1 is tumor SUV_max_–to–background SUV_max_ and TBR2 is tumor SUV_max_–to–background SUV_mean_; comparison of peritoneal metastasis locations between ^68^Ga-FAPI-04 PET/CT and laparoscopic staging (24); and analysis of net cost impact, calculated by comparing cost savings from avoiding unnecessary gastrectomies with ^68^Ga-FAPI-04 PET/CT versus laparoscopic staging.

### Cost Analysis

To assess the cost impact of ^68^Ga-FAPI-04 PET/CT and laparoscopic staging, 4 staging strategies were modeled in a theoretic decision tree to calculate the total costs of each strategy: no ^68^Ga-FAPI-04 PET/CT or laparoscopic staging (strategy 1), laparoscopic staging alone (strategy 2), ^68^Ga-FAPI-04 PET/CT alone (strategy 3), and the use of both ^68^Ga-FAPI-04 PET/CT and laparoscopic staging (strategy 4). The probability of positive or negative outcomes after ^68^Ga-FAPI-04 PET/CT and laparoscopic staging were calculated based on the observed frequencies. For each staging strategy, a decision-tree model depicted 2 alternative scenarios: gastrectomy and no gastrectomy. Patients without OPM or other metastases (negative outcome) were allocated to the gastrectomy branch, whereas those with OPM or other metastases (positive outcome) did not undergo gastrectomy. Direct costs of laparoscopic staging included surgery, anesthesia fees, pathology tests, postoperative-associated costs for 3 days, whereas the direct cost of gastrectomy included surgery, anesthesia fees, pathology tests, and postoperative-associated costs for 7 days. Average unit costs of ^68^Ga-FAPI-04 PET/CT, laparoscopic staging, gastrectomy, anesthesia fees, pathology tests, and hospitalization expenses (before insurance reimbursement) were obtained from the settlement list from West China Hospital in China. Cost analyses were performed using TreeAge Pro 2021 (TreeAge Software LLC).

### Statistical Analysis

Statistical analyses were performed using R version 4.2.3. The primary analysis was performed by the study biostatistician, and the report was reviewed by a second author for accuracy. The area under the curve was calculated based on sensitivity and specificity. Quantitative data were presented as mean ± SD if normally distributed or as median and interquartile range. Categoric variables are presented as numbers and percentages. Student *t* test and χ^2^ test were used to compare means and categoric variables, respectively. Fisher exact test was applied if the theoretic frequency was fewer than 5 in any group. The diagnostic performance of ^68^Ga-FAPI-04 PET/CT was assessed using sensitivity, specificity, positive predictive value, negative predictive value, and positive and negative likelihood ratios. Accuracy was calculated as the proportion of true positives and true negatives among all tested cases. Lesion detectability parameters, including SUV_max_ and TBR, were also calculated. A 2-tailed *P* value of less than 0.05 was defined as statistically significant.

## RESULTS

### Participant Characteristics

**FIGURE 1.**
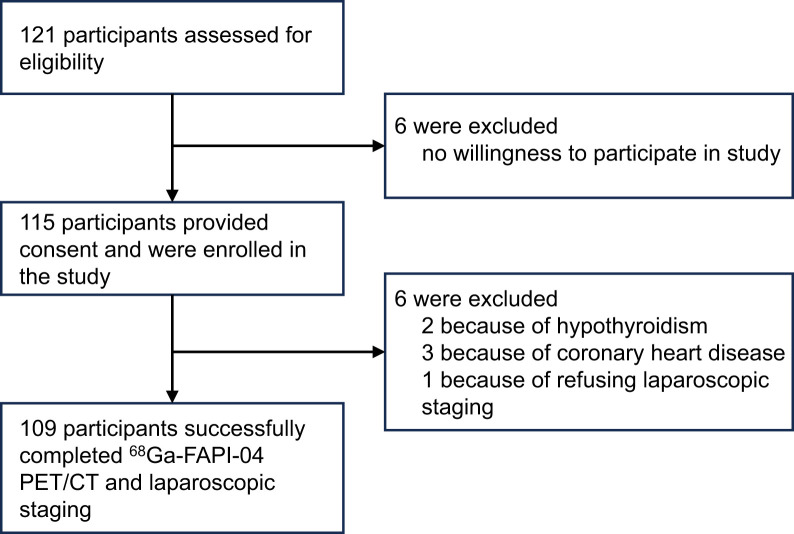
Study flow diagram.

In total, 121 patients were screened between November 2022 and August 2024, and 109 patients were enrolled (Fig. 1). Six patients were unwilling to participate in the study and therefore excluded. Another 6 patients did not undergo laparoscopic staging because of hypothyroidism (*n* = 2), coronary heart disease (*n* = 3), or refusal to undergo surgery (*n* = 1). Baseline characteristics of the study population are summarized in Table 1. Most tumors (*n* = 85; 78.0%) were located in the stomach, with the remaining 24 (22.0%) appearing in the esophagogastric junction (Siewert type III). Most primary tumors were staged as T4a (*n* = 100; 91.7%), with 9 (8.3%) staged as T4b. All patients (*n* = 109) underwent ^68^Ga-FAPI-04 PET/CT before laparoscopic staging and tolerated the examinations without complications.

**TABLE 1.** Baseline Characteristics of Study Population (*n* = 109)

Characteristic	Value
Age (y)	60.0 (51.5–69.0)
Sex	
Male	68 (62.4)
Female	41 (37.6)
Ethnicity	
Han	97 (89.0)
Minority	12 (11.0)
Height (cm)	165.0 (158.0–169.0)
Weight (kg)	60.0 (53.0–70.0)
BMI (kg/m^2^)	22.9 ± 3.4
Tumor location	
Esophagogastric junction	24 (22.0)
Gastric	85 (78.0)
Preoperative tumor staging	
cT4a	100 (91.7)
cT4b	9 (8.3)
Preoperative node staging	
cN0	37 (33.9)
cN1	40 (36.7)
cN2	21 (19.3)
cN3	11 (10.1)
Tumor marker value	
CEA (ng/mL)	
<5	87 (79.8)
≥5	23 (21.1)
CA125 (units/mL)	
<24	100 (91.7)
≥24	9 (8.3)
CA199 (units/mL)	
<30	89 (81.7)
≥30	20 (18.3)
CA72-4 (units/mL)	
<6.9	63 (57.8)
≥6.9	46 (42.2)
Pathologic pattern	
Signet ring cell carcinoma	32 (29.4)
Non–signet ring cell carcinoma	77 (70.6)
EBER-ISH	
Positive	7 (6.4)
Negative	51 (46.8)
Unknown	51 (46.8)
HER2	
0/1+	84 (77.1)
2+	15 (13.8)
3+	6 (5.5)
Unknown	4 (3.7)
MMR	
dMMR	11 (10.1)
pMMR	96 (88.1)
Unknown	2 (1.8)

BMI = body mass index; CEA = carcinoembryonic antigen; CA125 = carbohydrate antigen 125; CA199 = carbohydrate antigen 199; CA72-4 = carbohydrate antigen 72-4; EBER = Epstein-Barr virus-encoded RNA; ISH = in situ hybridization; HER2 = human epidermal growth factor receptor 2; MMR = mismatch repair; dMMR = deficient MMR; pMMR = proficient MMR.

Qualitative data are number and percentage. Continuous data are median and interquartile range or mean ± SD.

### ^68^Ga-FAPI-04 Uptake in Primary Tumors and Peritoneal Metastases

All patients exhibited a ^68^Ga-FAPI-04-avid primary tumor. As shown in Table 2, the mean SUV_max_ of ^68^Ga-FAPI-04 uptake in the primary tumor was 14.87 ± 6.12 across all enrolled patients. No significant difference in ^68^Ga-FAPI-04 uptake in the primary tumor was found between signet ring cell carcinoma and non–signet ring cell carcinoma (mean SUV_max_, 13.55 vs. 15.44, respectively; *P* = 0.145). The mean TBR1 and TBR2 of the primary tumor were 11.22 ± 4.89 and 15.69 ± 6.65, respectively. Patients with OPM exhibited significantly higher mean SUV_max_ and TBR2 values in the primary tumors compared with those without OPM (*P* = 0.010 and *P* = 0.009, respectively).

**TABLE 2.** ^68^Ga-FAPI-04 Uptake in Primary Tumor and OPM of Gastric Cancer

Variable	*n*	Tumor site	SUV_max_	TBR1	TBR2
All patients	109	Primary	14.87 ± 6.12	11.22 ± 4.89	15.69 ± 6.65
Patients without OPM	93	Primary	14.25 ± 5.95	10.87 ± 4.85	15.00 ± 6.36
Patients with OPM	16	Primary	18.49 ± 6.00	13.28 ± 4.78	19.65 ± 7.08
FAPI PET/CT and laparoscopy positivity	12	Peritoneum	5.93 ± 3.30	4.74 ± 2.66	6.33 ± 3.75
	12	Primary	15.13 ± 3.65	12.35 ± 3.50	16.23 ± 4.85

Background activities were determined from the mediastinal blood pool. Peritoneal tumor SUV_max_ was the average value of most active lesion from no more than 3 regions. Data represent mean ± SD.

### Diagnostic Performance of ^68^Ga-FAPI-04 PET/CT in OPM

Seventeen of 109 patients were diagnosed with OPM after ^68^Ga-FAPI-04 PET/CT. Laparoscopic staging with pathology confirmed 16 cases of peritoneal metastases: 7 with macroscopic (P1) and cytology-positive (CY1) disease; 6 with P1CY0, and 3 with P0CY1. Of all patients, 12 were diagnosed with OPM, whereas 88 were not found to have OPM by both ^68^Ga-FAPI-04 PET/CT and laparoscopic staging. As shown in Table 3, the sensitivity of ^68^Ga-FAPI-04 PET/CT for detecting OPM was 75.0%, and the specificity was 94.6%. The overall diagnostic accuracy of ^68^Ga-FAPI-04 PET/CT was 91.7%. For ^68^Ga-FAPI-04 PET/CT, the positive and negative predictive values were 70.6% and 95.7%, respectively. The positive and negative likelihood ratios of ^68^Ga-FAPI-04 PET/CT for the detection of OPM were 14.2 and 0.3, respectively. Receiver-operating-characteristic curve analysis yielded an area under the curve of 0.83 (95% CI, 0.72–0.94). There was a high concordance in OPM localization between ^68^Ga-FAPI-04 PET/CT and laparoscopic exploration, with 9 of 12 patients showing consistent localization (Supplemental Table 1).

**TABLE 3.** Performance of ^68^Ga-FAPI-04 PET/CT in Diagnosing OPM of Gastric Cancer

	Laparoscopic staging		
^68^Ga-FAPI-04 PET/CT	Positive	Negative	Total
Positive	12	5	17
Negative	4*	88	92
Total	16	93	109

### Two Typical Cases of Peritoneal Metastasis

**FIGURE 2.**
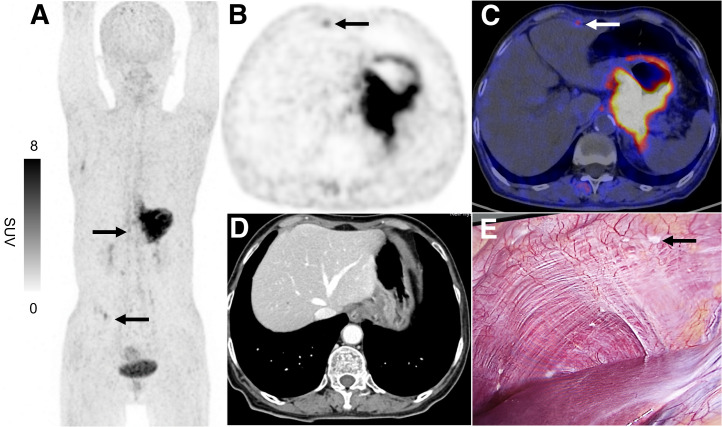
^68^Ga-FAPI-04 PET images of 71-y-old woman with poorly differentiated gastric adenocarcinoma and peritoneal carcinomatosis. (A) Maximum-intensity-projection image demonstrated multiple foci in upper and right lower abdominal quadrants (arrows; SUV_max_, 4.17). Axial PET (B) and PET/CT fusion (C) images localized upper abdominal focus to left subdiaphragmatic space (arrows), with no corresponding abnormality on contrast-enhanced CT (D). (E) Subsequent preoperative staging laparoscopy confirmed tiny implants on diaphragmatic surface (arrow).

**FIGURE 3.**
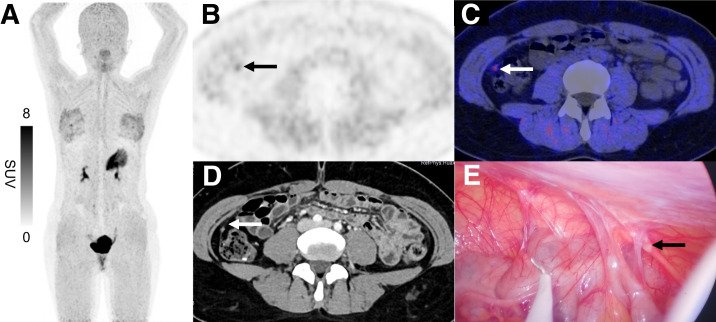
^68^Ga-FAPI-04 PET/CT images of 24-y-old woman with poorly differentiated gastric adenocarcinoma and peritoneal carcinomatosis. (A) Maximum-intensity-projection image showed no conspicuous abnormal tracer uptake in abdomen apart from primary tumor. However, axial PET (B) and PET/CT fusion (C) images revealed subtle focus in right paracolic gutter (arrows; SUV_max_, 1.71). (D) Corresponding contrast-enhanced CT demonstrated enhancing nodule that was initially missed. (E) Subsequent preoperative staging laparoscopy confirmed peritoneal implant at corresponding site (arrow).

Figures 2 and 3 display 2 typical images of peritoneal metastasis: 1 in the right upper quadrant of the abdomen and the other in the right paracolic gutter. No abnormalities were detected on the corresponding enhanced CT scan at the initial diagnosis. However, a notable increase in ^68^Ga-FAPI-04 uptake was observed on ^68^Ga-FAPI-04 PET/CT examination. Subsequent laparoscopic staging verified that the presence of tiny implants at the corresponding site was peritoneal metastasis.

### Changes in Tumor Staging After ^68^Ga-FAPI-04 PET/CT

**FIGURE 4.**
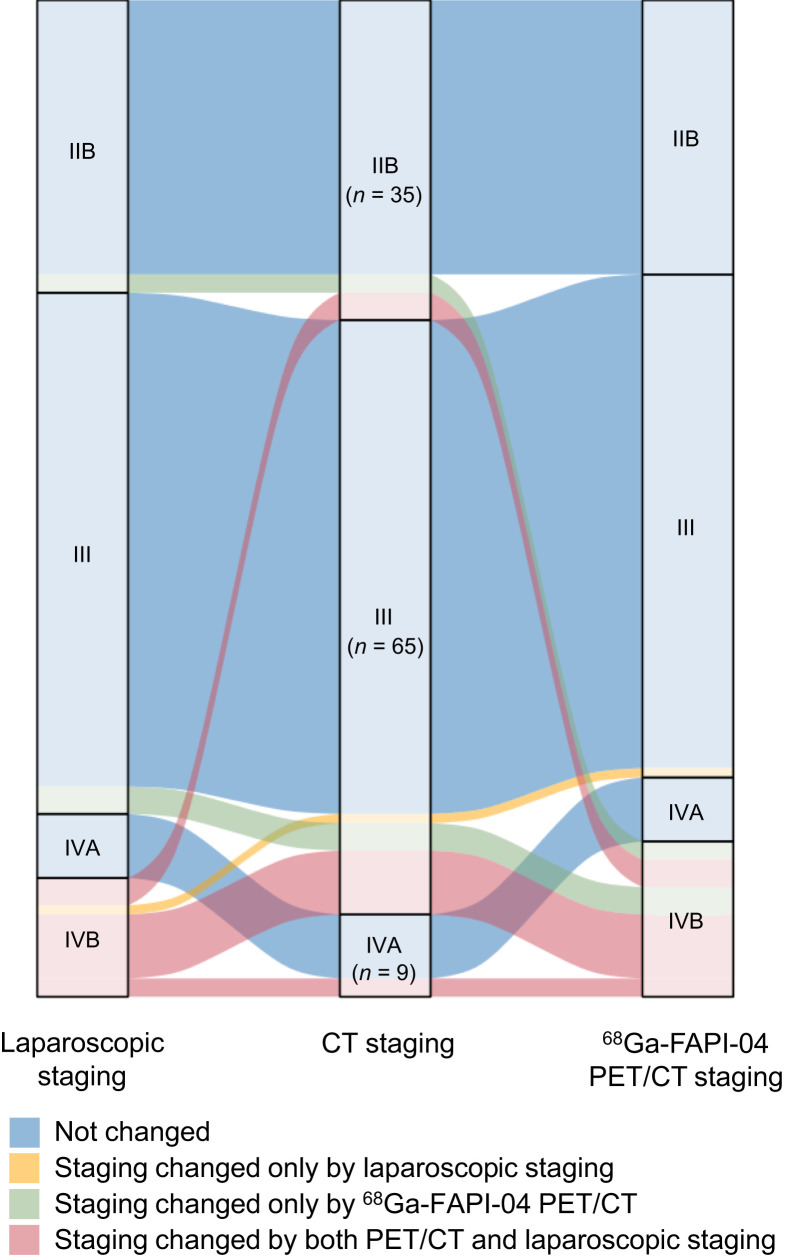
Visualization of inner layers of staging showed metastatic staging of patients changed after FAPI PET/CT examination and laparoscopic staging. Nodes on far left represent metastatic staging changed by laparoscopic staging. Nodes in second layer represent initial staging based on CT. Final layer represents metastatic staging changed by FAPI PET/CT. Staging changed only by FAPI PET/CT in 17 patients(15.6%), of whom 5 (4.6%) changed from IIB to IVB, 10 (9.2%) changed from III to IVB, and 2 (1.8%) changed from IVA to IVB. Of these 17 cases, 12 (70.6%) were identified as peritoneal metastases by laparoscopic staging. Only positive peritoneal cytology (CY1) was found in 3 of 17 patients.

Seventeen patients (15.6%) were theoretically upstaged to stage IV for OPM on ^68^Ga-FAPI-04 PET/CT examination; no other distant metastases were detected in any patient (Fig. 4). In fact, treatment intent changed from curative to palliative in 12 of these 17 patients. For the remaining 5 patients, OPM was not confirmed by laparoscopic staging or peritoneal cytology, so they were still treated with curative intent. As shown in Figure 4, 2 patients initially staged as IIB and 3 patients initially staged as III experienced an upstaging after ^68^Ga-FAPI-04 PET/CT diagnosis, but their staging remained unchanged after laparoscopic exploration. Conversely, 4 patients initially staged as III were upstaged to IV after laparoscopic exploration, whereas their staging remained unchanged after ^68^Ga-FAPI-04 PET/CT examination.

### Cost Analysis

**FIGURE 5.**
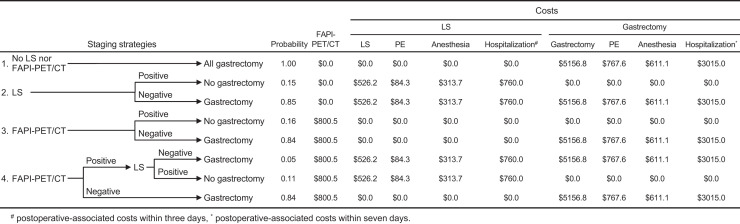
Decision tree to assess the cost impact of laparoscopic staging and ^68^Ga-FAPI-04 PET/CT by modelling four staging strategies.

Figure 5 presents a decision tree analyzing the cost impact of laparoscopic staging and ^68^Ga-FAPI-04 PET/CT across 4 modeled staging strategies. In a theoretic model where laparoscopic staging alone was performed preoperatively for patients with locally advanced gastric cancer, 16 futile gastrectomies would have been performed (strategy 2). Conversely, if only ^68^Ga-FAPI-04 PET/CT was performed preoperatively, 17 futile gastrectomies would have been performed (strategy 3). Based on diagnostic performance of ^68^Ga-FAPI-04 PET/CT, we proposed performing laparoscopic staging only for ^68^Ga-FAPI-04–positive patients and avoiding it for ^68^Ga-FAPI-04–negative patients (strategy 4). When no peritoneal abnormalities were detected on ^68^Ga-FAPI-04 PET/CT, 84.0% (*n* = 92) of patients could potentially avoid laparoscopic staging. Conversely, in cases where high ^68^Ga-FAPI-04 uptake was observed, laparoscopic staging would be performed in 17 patients (16%) as a supplemental method to detect peritoneal metastasis. Using this strategy, the number of futile gastrectomies would be reduced to 12. If all patients underwent gastrectomy without ^68^Ga-FAPI-04 PET/CT and laparoscopic staging (strategy 1), the total cost would be $9,550.50 per patient. Staging with ^68^Ga-FAPI-04 PET/CT (strategy 3) reduced costs by $727.60 per patient, whereas laparoscopic staging (strategy 2) increased costs by $251.70 per patient. Compared with laparoscopic staging (strategy 2), ^68^Ga-FAPI-04 PET/CT (strategy 3) achieved a net cost savings of $979.30 per patient. To enhance the clinical applicability of the theoretic strategies, we developed a new approach (strategy 4) based on the higher negative predictive value of ^68^Ga-FAPI-04 PET/CT. If no peritoneal abnormalities were detected by ^68^Ga-FAPI-04 PET/CT, laparoscopic staging could be omitted; however, if significant uptake was observed in the peritoneum, laparoscopic staging was required to confirm peritoneal metastases. This strategy resulted in a minimal net cost savings of $232.30 per patient compared with laparoscopic staging (strategy 2), while significantly reducing the need for laparoscopic staging by 84% and preventing 11% of futile gastrectomies.

## DISCUSSION

In our study, ^68^Ga-FAPI-04 PET/CT demonstrated a sensitivity of 75.0% and a specificity of 94.6% in detecting OPM, with an overall diagnostic accuracy of 91.7%. Notably, the negative predictive value reached 95.7%, allowing clinicians to confidently exclude OPM in clinical practice. These findings highlight the significant advantages of ^68^Ga-FAPI-04 PET/CT and emphasize its role as a standard method for detecting OPM in patients with locally advanced gastric cancer.

By targeting the tumor stroma, which constitutes most of the tumor volume, ^68^Ga-FAPI-04 PET/CT is considered more sensitive for detecting small tumor lesions (25). Compared with ^18^F-FDG PET/CT, ^68^Ga-FAPI-04 PET/CT demonstrated superior sensitivity in detecting peritoneal metastases, particularly in gastrointestinal and ovarian cancers, because of its minimal physiologic intestinal uptake, thereby providing more accurate guidance for treatment decisions (14,26–29). However, existing studies evaluating ^68^Ga-FAPI-04 PET/CT for detecting peritoneal metastases have notable limitations. Most were retrospective in design, included heterogeneous cohorts of gastrointestinal malignancies with small sample sizes (27,30), and often lacked laparoscopic staging as the reference standards. These methodologic constraints limit the robustness and generalizability of their findings (17,31,32). Even the limited prospective studies in gastric cancer assessed all peritoneal metastases rather than specifically addressing OPM, and subgroup analyses further inflated detection rates (16,33). To our knowledge, this prospective cohort study is the first to adopt laparoscopic staging as the reference standard for evaluating the diagnostic value of ^68^Ga-FAPI-04 PET/CT in detecting OPM in gastric cancer.

The false-positive rate of ^68^Ga-FAPI-04 PET/CT cannot be overlooked, as 5 false-positive lesions were identified in our study. One possible explanation is the false-negative findings from laparoscopic staging, which has a reported false-negative rate of 2.8%–17.2% (8,34–38), were potentially influenced by whether patients underwent open bursa omentalis surgery. Although peritoneal metastasis in the transverse mesocolon and omentum majus is a concern, the necessity of visualizing the bursa omentalis during laparoscopic exploration remains controversial (34,39). In our study, we used a 4-step procedure that does not include routine visualization of the bursa omentalis during laparoscopic staging. One false-positive ^68^Ga-FAPI-04 PET/CT case showed abnormal activity on the pancreas surface, a blind spot in laparoscopic staging (Supplemental Fig. 1). This finding highlights the need for further investigation into the procedure and scope of laparoscopic staging. Additionally, we speculate that chronic inflammation combined with fibrosis may contribute to false-positive results.

The false-negative rate of ^68^Ga-FAPI-04 PET/CT is a critical consideration for its clinical application. In our study, 4 false-negative lesions were observed on ^68^Ga-FAPI-04 PET/CT, with 3 patients diagnosed as having positive peritoneal cytology (CY1) only. Retrospective studies have reported that 12.5% of patients had positive peritoneal cytology in the absence of macroscopic metastases (40). Similarly, a prospective study found that 0.6% of patients with no or low-level clinically suspicious lesions and 5.8% of patients with suspicious peritoneal lesions were diagnosed with peritoneal metastasis based on positive peritoneal cytology (P0 and CY1) (22). In our study, 2.8% of patients without macroscopic metastases were found to have positive peritoneal cytology, highlighting the limited ability of ^68^Ga-FAPI-04 PET/CT in detecting CY1. Furthermore, small-volume tumors may be underestimated because of partial-volume effects, low metabolic activity, and the limited resolution of PET imaging, all of which could contribute to the false-negative rate of ^68^Ga-FAPI-04 PET/CT (41,42).

The detection rate of OPM using PET/CT is critical for economic evaluations. Routine ^18^F-FDG PET/CT is not recommended because of its high costs and limited effectiveness, reducing the rate of unnecessary gastrectomies by only 3% (43). When the detection rate of occult metastatic disease was 10%, the addition of ^18^F-FDG PET/CT resulted in an estimated cost savings per patient with locally advanced gastric cancer but failed to identify any peritoneal metastases in the study (44). In contrast, our study found that ^68^Ga-FAPI-04 PET/CT, when compared with laparoscopic staging, prevented futile gastrectomies in nearly 16% of patients, resulting in a potential savings of $565.30 per patient. Given the high negative predictive value (95.7%) of ^68^Ga-FAPI-04 PET/CT for diagnosing OPM in our study, we proposed an optimized diagnostic workflow (strategy 4) for patients with locally advanced gastric cancer in which patients first undergo ^68^Ga-FAPI-04 PET/CT. If no peritoneal abnormalities are observed, laparoscopic staging can be omitted. If high ^68^Ga-FAPI-04 uptake is detected in the peritoneum, laparoscopic staging will be performed to confirm peritoneal metastases. This strategy is economically comparable to laparoscopic staging but aims to reduce unnecessary laparoscopic staging and gastrectomies by relying on ^68^Ga-FAPI-04 PET/CT findings.

This study had several limitations. First, this was a single-center prospective study with a relatively small number of enrolled patients. Future studies involving larger cohorts and multicenter prospective designs would provide a more robust evaluation of the diagnostic efficacy of ^68^Ga-FAPI-04 PET/CT. Second, we did not compare ^68^Ga-FAPI-04 PET/CT with ^18^F-FDG PET/CT in randomized controlled trials, primarily because of the well-recognized limitations of ^18^F-FDG PET/CT in diagnosing gastric cancer, particularly in detecting OPM. Third, this study focused exclusively on patients with advanced gastric cancer (cT4a–b, N0–3, M0) based on initial staging evaluations using contrast-enhanced CT. However, CT-based diagnoses can be subjective and operator-dependent, potentially introducing variability. Lastly, we did not perform immunohistochemical analyses in this study because of the limited availability of tissue, despite the fact that it is the most direct and rigorous method to determine whether fibroblast activation protein expression originates from tumor cells or cancer-associated fibroblasts within the stroma.

## CONCLUSION

The results of this study highlight the added value and minimal risks of using ^68^Ga-FAPI-04 PET/CT for detecting OPM. As a promising, precise, and rapid staging tool for locally advanced gastric cancer, ^68^Ga-FAPI-04 PET/CT is particularly valuable for excluding OPM as a diagnoses. Nonetheless, further high-quality, large-scale clinical trials are needed to validate these findings before implementing changes in clinical practice or guidelines.

## DISCLOSURE

This work was supported by the Key R&D Program of Sichuan Provincial Department of Science and Technology (No. 2024YFFK0341), 1.3.5 project for disciplines of excellence, and the Resident/Specialist Research Fund of the West China Hospital, Sichuan University. No other potential conflict of interest relevant to this article was reported.

## Notes

KEY POINTS **QESTION:** Does ^68^Ga-FAPI-04 PET/CT show promise for detecting OPM in locally advanced gastric cancer? **PERTINENT FINDINGS:** In a single-center, prospective cohort of 109 patients with locally advanced gastric adenocarcinoma, ^68^Ga-FAPI-04 PET/CT detected OPM in 17 patients (15.6%), resulting in upstaging to stage IV. The modality demonstrated robust diagnostic performance for identifying OPM, with a sensitivity of 75.0%, a specificity of 94.6%, and an overall accuracy of 91.7%. **IMPLICATIONS FOR PATIENT CARE:** ^68^Ga-FAPI-04 PET/CT offers high diagnostic accuracy for detecting OPM, supporting its use as a promising alternative to laparoscopic staging in locally advanced gastric cancer.
